# Effect of ionic liquid on formation of copolyimide ultrafiltration membranes with improved rejection of La^3+^

**DOI:** 10.1038/s41598-022-12377-0

**Published:** 2022-05-17

**Authors:** Alexandra Pulyalina, Konstantin Grekov, Vera Tataurova, Anna Senchukova, Alexander Novikov, Ilya Faykov, Galina Polotskaya

**Affiliations:** 1 Institute of Chemistry, Saint Petersburg State University, Saint Petersburg, 198504 Russian Federation; 2 The Bonch-Bruevich St. Petersburg State University of Telecommunications, Saint Petersburg, 193232 Russian Federation; 3 Institute of Macromolecular Compounds, Russian Academy of Sciences, Saint Petersburg, 199004 Russian Federation

**Keywords:** Ecology, Materials science, Chemistry, Green chemistry

## Abstract

Ultrafiltration (UF) as a widely used industrial separation method with optimal selection of membrane materials can be applied to extract rare earth metals from dilute solutions formed during the processing of electronic waste by hydrometallurgical methods. In the present work, promising UF copolyimide membranes were prepared using [hmim][TCB] ionic liquid (IL) co-solvent which can be considered as an environmentally friendly alternative to conventional solvents. The membranes were characterized by ATR-FTIR, TGA, SEM and quantum chemical calculations. A significant difference in morphology of these membranes was revealed by SEM of membrane cross-sections; the P84 membrane has finger-like structure of porous substrate in contrast to spongy structure of substrate for the P84/IL membrane due to a higher dynamic viscosity of the casting solution. The transport parameters were determined in ultrafiltration tests with pure water and an aqueous solution of bovine serum albumin. The addition of ionic liquid to the P84 casting solution increases the performance of the membrane. The rejection capacity was evaluated with respect to La^3+^ in the form of a lanthanum alizarin complex (LAC) in aqueous acetone solution. The P84 membrane prepared using IL showed a high rejection (98.5%) with respect to LAC, as well as a significant productivity.

## Introduction

Membrane techniques with their great advantages over conventional separation methods are effectively used to solve urgent environmental and industrial tasks of purifying aqueous solutions and separating components from liquid media^1–5^. Membrane techniques require minimal energy and economic costs, they are easy to operate, implement and maintain. Modern ultrafiltration (UF) is constantly developed and finds its application in food, pharmaceutical, textile, paper, and other industries^6–8^. Large-scale processes, such as high-temperature filtration of viscous oils, water purification in nuclear reactors, chemical catalysis, gas-phase reactions and others, require UF membranes that exhibit not only high permeability, selectivity and good mechanical properties, but also thermal and chemical stability^9–12^. Among commercially available materials, polymers with imide units in the backbone possess the above properties to a large extent, which is due to the presence of rigid heterocyclic and aromatic rings in their chains^13–23^. P84 copolyimide (BTDA-TDI/MDI) is a product of polycondensation between benzophenone-3,3',4,4'-tetracarboxylic dianhydride (BTDA) and 2,4-tolylene diisocyanate/1,1′-methylenebis(4-isocyanatobenzene) (80/20%) ; this commercially available polyheteroarylene exhibits good mechanical properties, chemical resistance, and low hydrophilicity. P84 copolyimide has been studied as a membrane material for nanofiltration^24^, gas separation^25,26^, and pervaporation^14,27–30^.

The study on P84 membranes in UF processes has been carried out in^31–34^. In the work^34^, the authors compared the structure and properties of the membranes prepared from three commercial polyimides: P84, Matrimid, and Torlon depending on the used solvent, N-methylpyrrolidone (NMP) or dimethylsulfoxide (DMSO). Membranes were formed by phase inversion technique using water as a coagulant. The P84 membrane obtained from DMSO solution had a spongy porous structure, in contrast to the finger-like structure of the membrane obtained from NMP solution. The difference in the structures was reflected in the transport properties of the UF membranes. It was found that the P84 membrane obtained from a solution in NMP possessed the best transport properties.

Polymer membranes are commonly prepared from solutions based on polar aprotic solvents, such as NMP, DMSO, dimethylacetamide, and dimethylformamide due to their chemical affinity for a polymer; the listed polar solvents are highly toxic to human health and ecosystems. Ionic liquids (IL) provide an environmentally friendly alternative to conventional solvents^35^; IL are organic salts that remain liquid at room temperature, have good thermal and chemical properties, low volatility and can be recycled and reused. The use of such “green” solvents would minimize waste and losses in chemical processes^36^. It has been shown in^37^ that the use of 1-ethyl-3-methylimidazolium tetrafluoroborate as an additive (up to 17% IL) in the polyethersulfone solution in DMF for the formation of UF membranes leads to a significant increase in the flux for an aqueous solution of bovine serum albumin and in the flux recovery ratio.

Comparison of cellulose acetate (CA) membranes obtained from 10 wt% CA solution of either 1-butyl-3-methylimidazolium thiocyanate (IL) or NMP by precipitation into water showed that the CA/IL membrane had very low porosity ~ 6% and reduced performance in contrast to CA/NMP membrane with porosity ~ 84%^38^. According to the SEM data, the CA/NMP membrane had more open pores than the CA/IL membrane. The effect of IL depends also on the type of polymers, even such similar as cellulose and CA^39^. The addition of IL (1-ethyl-3-methylimidazolium acetate) in an amount from 0 to ~ 50 wt% to the casting solution of 8 wt% cellulose in DMSO had almost no effect on the membrane structure and properties. However, similar IL addition to the casting solution of CA in DMSO led to a change in the membrane morphology, an increase in the flux, but to a decrease in the rejection of dextran blue.

**Figure 1:**
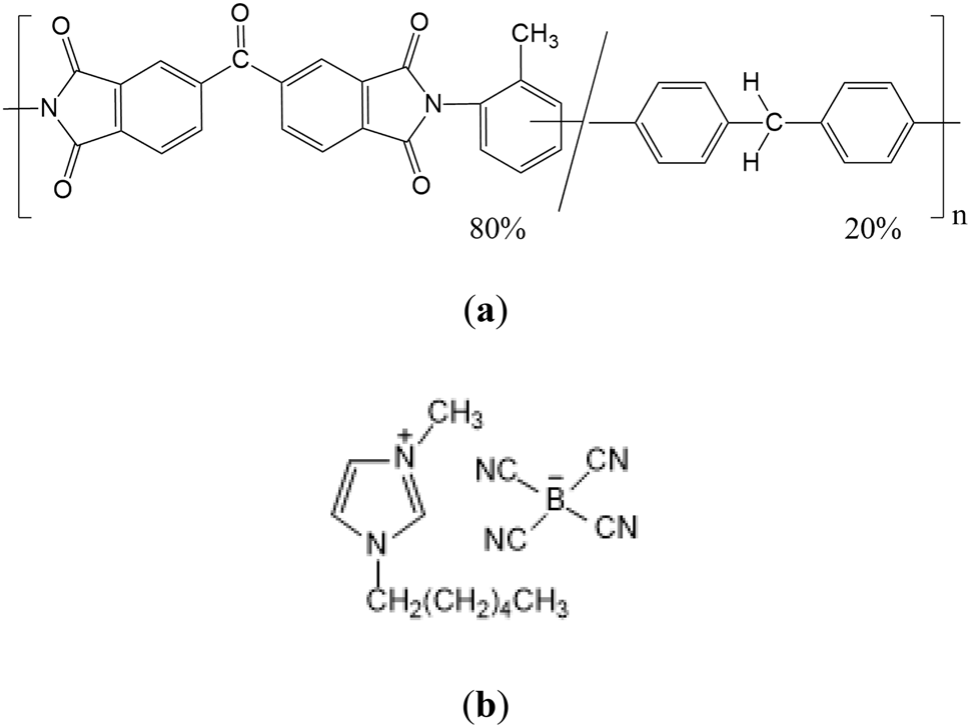
Structural formulas of (**a**) P84 copolyimide and (**b**) 1-hexyl-3-methylimidazolium tetracyanoborate (IL).

The aim of the present work was to study the effect of 1-hexyl-3-methylimidazolium tetracyanoborate—[hmim][TCB] (IL) additives on the structure and transport properties of UF membranes prepared from P84 copolyimide (Fig. 1). Another important task was to assess the possibility of using these membranes for the rejection of La^3+^. It is known that the method of reagent ultrafiltration can be applied to extract rare earth metals from dilute solutions formed during the processing of electronic waste by hydrometallurgical methods^40–42^. The greatest interest is in the implementation of this method by the formation of complex ions that are larger than the initial ions of the isolated metal by introducing a specially selected complexing agent. In this work, in order to assess the rejection capacity of the P84 and P84/IL membranes with respect to La^3+^, we used alizarin chelator which forms a complex compound with La^3+^, i.e., lanthanum-alizarin complex (LAC) in aqueous acetone solution^43,44^.

## Materials and methods

### Materials

*N*-methylpyrrolidone (NMP) of analytical grade and potassium phosphate of high-purity grade were purchased from Spectr-Chem (St. Petersburg, Russia). 1-hexyl-3-methylimidazolium tetracyanoborate of HPLC grade (≥ 97%) ([hmim][TCB]) was purchased from Merck (Darmstadt, Germany). Commercial P84 powder was purchased from HP Polymer GmbH (Lenzing, Austria). Lanthanum nitrate hexahydrate of chemically pure grade was purchased from ChemExpress (St. Petersburg, Russia). Sodium chloride and sodium phosphate 2-substituted of high-purity grade were purchased from Vekton (St. Petersburg, Russia). Bovine serum albumin of HPLC grade (≥ 99%) was purchased from Dia-M (Moscow, Russia); bovine γ-globulin and ovalbumin of HPLC grade (≥ 97%) were purchased from Sigma-Aldrich Chemie GmbH. (Sigma-Aldrich, Schnelldorf, Germany); vitamin B12 of HPLC grade (≥ 98%) was purchased from J&K Sientific Ltd. (San Jose, USA).

### Preparation of membranes

Asymmetric membranes were obtained from casting solutions of P84 in NMP or from a solution of P84/IL/NMP in the ratio 15/15/70 (wt%). The casting solution was poured onto a glass plate using a casting knife with a nominal thickness of 300 μm, and the glass plate was immersed immediately into a coagulating bath with water/ethanol mixture in the ratio 60/40 (wt%) at room temperature. The asymmetric porous membrane was formed as a result of the phase inversion process; it was kept in the coagulating bath for ~ 3 h. Then the membrane was washed three times with an aqueous solution of ethanol, hexane and dried.

The dynamic viscosity of P84 and P84/IL casting solutions in NMP was measured using a vibration viscometer SV-10A, A&D (Japan) with a measuring range of 0.3–10,000 mPa s. The measurement was carried out at 25 °C.

### Computational details

The full geometry optimization of all model structures was carried out at the DFT level of theory using the dispersion-corrected hybrid functional ωB97XD^45^ with the help of Gaussian-09 program package^46^. The standard 6-31G* basis sets were used for all atoms. No symmetry restrictions have been applied during the geometry optimization procedure. The Hessian matrices were calculated analytically for optimized model structures to prove the location of correct minima on the potential energy surfaces (no imaginary frequencies were found in all cases). The thermodynamic parameters were calculated at 298.15 K and 1.00 atm. The enthalpies, entropies, and Gibbs free energies of optimized equilibrium model structures are presented in Tables S1. Detailed description of enthalpies, entropies, and Gibbs free energies calculations procedure is given in^22^.

### Membrane characterization

Ultrafiltration tests were carried out in the dead-end cell with a membrane diameter of 25 mm equipped with a stirrer with a speed of 200**–**600 rpm, a transmembrane pressure was maintained at ~ 1 bar by nitrogen flow^47^. The filtrate (permeate) amount was determined using electronic balance.

Membrane calibration was carried out according to the technique described in^48^. UF test was performed using 1 wt% mixture of proteins with different molecular weight (Table 1) in a phosphate buffer solution, pH  7. The concentration of the proteins in the feed and permeate was analyzed using a PE-5400UF spectrophotometer (Ekroschem, St. Petersburg, Russia). The measurements were carried out at a wavelength of 280 nm since it is the maximum absorption of the selected proteins.

**Table 1:** Proteins for membrane calibration.

Protein	Molecular weight, g/mol	Stokes radius*,* Å
Vitamin B_12_	1360	7.8
Ovalbumin	44,000	28.6
Bovine serum albumin	67,000	34.0
γ-globuline	160,000	46.5

To estimate separation efficiency of membranes toward La^3+^, a solution of lanthanum—alizarin complex (LAC) was prepared from the following components: 2 cm^3^ of an acetate buffer solution, 10 cm^3^ of a lanthanum nitrate solution with a concentration of 5 mmol/dm^3^, 10 cm^3^ of an alizarin complexone solution (5 mmol/dm^3^) and 25 cm^3^ of acetone. Being thorough mixed, 20 cm^3^ of the prepared solution was taken with a calibrated pipette and transferred to a volumetric flask of 100 cm^3^.

The concentration of LAC in the permeate and feed was determined using a KFK-ZKM photoelectric concentration colorimeter (Unico-Sis, St. Petersburg, Russia) (a light filter with a maximum transmission of 590 ± 10 nm and a bandwidth of 25 ± 10 nm).

The data of UF experiments were used to calculate the transport properties of the membranes.

The flux through the membranes, *J* (L m^−2^ h^−1^ bar^−1^), was calculated as:

1 $$J=\frac{V}{t\cdot S\cdot P}$$

where *V* is the volume of the permeate, L; *t* is the filtration time, h; *S* is the membrane surface area, m^2^; and *P* is the transmembrane pressure, bar.

The rejection (*R*) was calculated as:

2 $$R\,=\,\left(1- \frac{{C}_{p}}{{C}_{0}}\right)\,\times\,100\,\%$$

where *C*_*p*_ and *C*_*0*_ are the protein (or other rejected component) concentrations in the permeate and feed, respectively, g/L.

The flux recovery ratio (*FRR*) was calculated using the following equation:

3 $$FRR = \frac{{J}_{0t}}{{J}_{0}}$$

where *J*_*0*_ is the pure water flux through the membrane, *J*_*0t*_ is the pure water flux after ultrafiltration of the protein solution at the same pressure.

The dispersion of rejection (*σ*) was calculated as:

4 $$  \sigma = 0.895 lg \, (M_{0.9} /M_{0.1} ) $$

where *M*_*0.9*_ and *M*_*0.1*_ are the molecular weights of the proteins that were rejected by the membrane on 90% and 10%, respectively.

To determine membrane porosity, a membrane was immersed in distilled water for 12 h, then weighed after removing excess moisture from the surface using filter paper. Further, the membrane was placed in a vacuum oven, dried for 12 h at 60 °C, and weighted. All weight measurements were performed using a Mettler Toledo analytical balance (with an error ± 0.0001 g). The membrane porosity was calculated using the Eq. (5)^49^:

5 $$P=\frac{{W}_{w}- {W}_{d}}{{\rho }_{w}Sd}$$

where *W*_*w*_ and *W*_*d*_ are the weights of the wet and dry membrane, respectively, g; *ρ*_*w*_ is the water density (0.998 g/cm^3^); *S* is the membrane area, cm^2^; *d* is the average thickness of the membrane, cm.

Membrane morphology was studied by scanning electron microscope SEM Zeiss SUPRA 55VP (Carl Zeiss AG, Germany). For obtaining cross-sectional micrographs, the membrane samples were pretreated with liquid nitrogen. Before the test the platinum layer of 20 nm thick was coated on the sample surface by cathode sputtering using the Quorum 150 (Great Britain) installation.

Thermogravimetric analysis (TGA) was carried out on a TG 209 F1 analyzer (Netzsch, Germany) using samples weighing ~ 8–15 mg under conditions of a dynamic temperature increase from 40 to 420 °C in a nitrogen atmosphere.

ATR-FTIR spectra of the membranes were recorded on IR-Fourier spectrometer Bruker Tensor 27 (Bruker Daltonics, Billerica, Massachusetts, Germany) with a resolution of 1 cm^−1^ within the range of 4000–500 cm^−1^ at ambient temperature (25 °C).

The Hildebrand solubility parameter (*δ*) was calculated as^50^:

6 $$\delta\,=\, {\left(\frac{{\Delta\,E}_{w}}{{V}_{w}}\right)}^{1/2}$$

where *∆E*_*w*_*/V*_*w*_ is the cohesion energy density required to overcome all intermolecular forces within 1 cm^3^ of a substance.

## Results and discussion

### Membrane formation option

**Figure 2:**
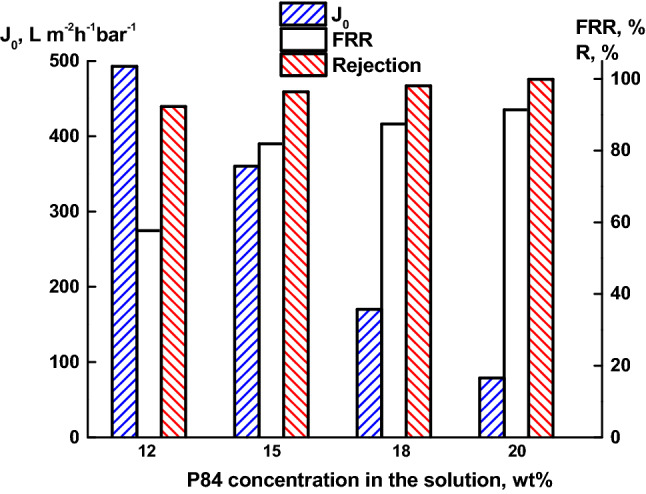
Dependence of the pure water flux (*J*_*0*_), rejection (*R*) and the flux recovery ratio (*FRR*) of P84 membranes on the P84 concentration in the casting solution.

The selection of the conditions for forming membranes of an asymmetric structure based on P84 was carried out by varying the polymer concentration in the NMP solution. Figure 2 shows the data on the pure water flux (*J*_*0*_), the rejection (*R*) of bovine serum albumin (BSA), as well as the flux recovery ratio (*FRR*) depending on the P84 concentration in the casting solution. As the polymer concentration increases, the flux decreases, but the *FRR* and rejection increase. This is due to the fact that an increase in the polymer concentration leads to an increase in the casting solution viscosity, which slows down the diffusion of the coagulant and, thus, the rate of phase inversion decreases. As a result, membranes with a denser selective layer and smaller pore sizes are formed^51^. A smaller pore size determines a decrease in the flux, but also a decrease in the adsorption of protein molecules on their surface, which contributes to an increase in *FRR*.

**Figure 3:**
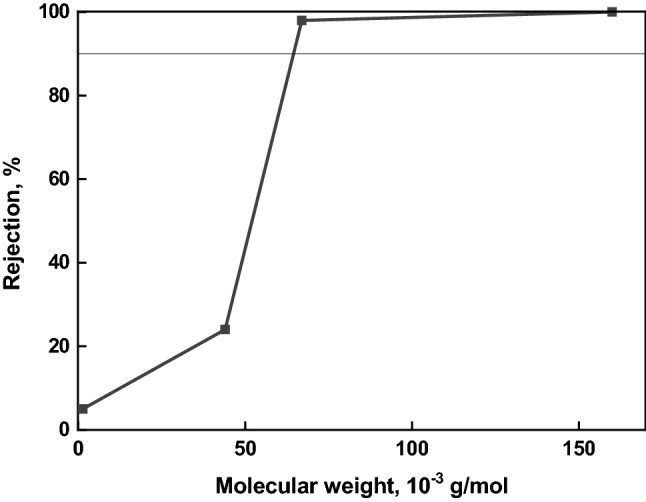
Dependence of protein rejection on the molecular weight for the P84 membrane prepared from a 15 wt% solution.

The optimal combination of the transport properties (*J*, *R*, *FRR*) was obtained for the membrane prepared from a 15 wt% P84 solution. To assess the separation efficiency of the membrane prepared from 15 wt% P84, UF calibration tests were performed using a 1 wt% aqueous solution of a mixture of four proteins differing in molecular weight (Table 1). Figure 3 shows the dependence of the rejection on the protein molecular weight. This curve was used to determine the value of the molecular weight cut-off (*MWCO*) that corresponds to the weight of the protein rejected by 90%^48,52^.

The *MWCO* for this P84 membrane is 65 000 g/mol. Figure 3 was also used to assess dispersion (*σ*) of the retention curve which was calculated by Eq. (4). For the developed asymmetric membrane this parameter was equal to ~ 1.4. Typically, the dispersion of conventional polymer membranes is in the range of 0.7–1.5. The latter can completely separate components that differ in molecular weights by tens and hundreds times.

The porosity of the P84 membrane prepared from a 15 wt% solution was determined using Eq. (5). This value is about 65% which correlates with the parameters of industrial membranes^49^.

### Membrane features

To assess the effect of IL additives on the structure and properties of asymmetric membranes, the P84/IL membranes were prepared from a casting solution containing P84/IL/NMP in the ratio 15/15/70 (wt%). The 1-hexyl-3-methylimidazolium tetracyanoborate IL was used as a co-solvent for NMP. Due to the presence of the imidazolyl cation in the IL structure, good compatibility of IL occurs with both P84 copolyimide and NMP solvent.

Table 2 lists the properties of the liquids used as a solvent. The Hildebrand solubility parameters for IL and NMP are close, which contributes to their affinity and good compatibility with the membrane polymer, since the Hildebrand solubility parameter for P84 is 26.4 (J/cm^3^)^1/2^.

**Table 2:** Physical properties of solvents.

Liquid	NMP	IL^53^
Property		
Molecular weight, g/mol	99	282
Density, g/cm^3^	1.028	0.991
Viscosity, mPa s	1.66	47.80
Hildebrand solubility parameter, δ, (J/cm^3^)^1/2^	22.9	21.3

Assumptions of the good compatibility of IL with NMP and P84 were confirmed by quantum chemical calculations. Interactions of amide solvent, IL and copolyimide were considered as hypothetical supramolecular association processes. The model reactions are presented in Table 3.

**Table 3:** Calculated values of enthalpies and Gibbs free energies of reaction (ΔH and ΔG) for various hypothetical supramolecular association processes.

Supramolecular association process	ΔH, kcal/mol	ΔG, kcal/mol
Ionic_liquid_cation + *N*-methylpyrrolidone → ionic_liquid_cation---*N*-methylpyrrolidone	−21.0	−10.0
Ionic_liquid_anion + *N*-methylpyrrolidone → ionic_liquid_anion---*N*-methylpyrrolidone	−7.4	2.1
P84 + *N*-methylpyrrolidone → P84---*N*-methylpyrrolidone	−12.9	0.7
P84 + ionic_liquid_cation → P84---ionic_liquid_cation	−24.5	−8.7
P84 + ionic_liquid_anion → P84---ionic_liquid_anion	−17.0	−5.0

**Figure 4:**
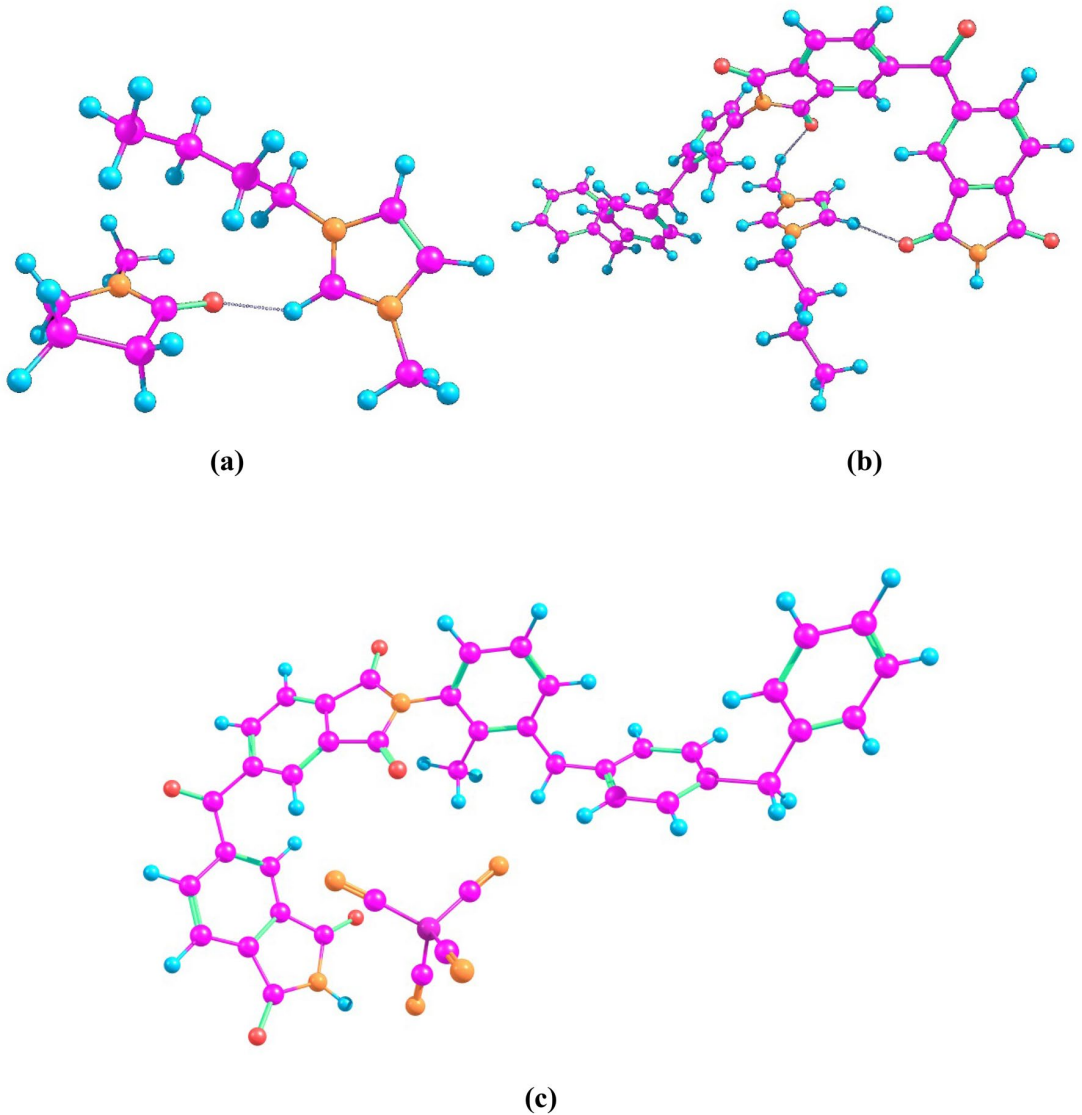
Scheme of coordination of (**a**) IL cation with NMP, (**b**) IL cation with P84, and (**c**) IL anion with P84.

The results of quantum chemical calculations (Table 3 and Table S1) reveal that all the studied hypothetical supramolecular association processes are exothermic. In terms of Gibbs free energies, the most thermodynamically favorable model reactions are associations of IL cation with NMP as well as IL cation and anion of with copolyimide P84 (Fig. 4). Thus, it can be concluded that NMP and IL are thermodynamically stable and IL can be used as relevant cosolvent for P84.

The IL addition promotes the formation of more viscous polymer solutions. The dynamic viscosity of 15 wt% P84/IL casting solution is equal to 6.33 P s while this parameter for 15 wt% P84 casting solution is only 1.89 P s. Thus, IL addition increases the viscosity of the polymer casting solution by more than three times. An increase in the viscosity of the polymer casting solution significantly affects the process of P84/IL membrane formation using phase inversion technique.

**Figure 5:**
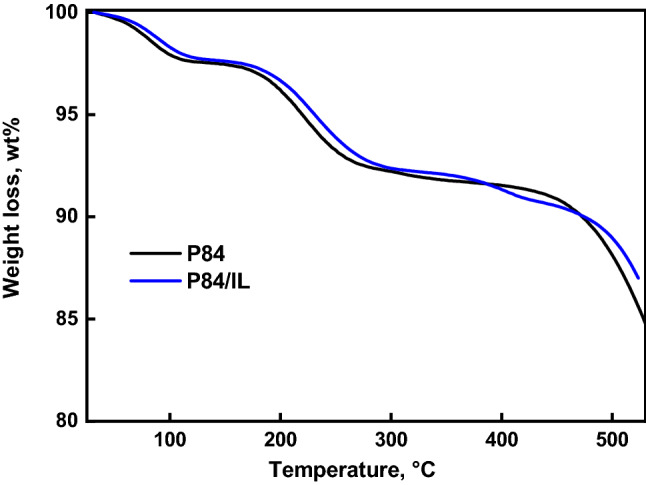
TG curves of the P84 and P84/IL membranes.

The thermal stability of the membranes was studied by thermogravimetric analysis in an inert atmosphere. Figure 5 shows TG curves for P84 and P84/IL membranes. The weight loss pattern is identical for the both membranes. The first range of weight loss ~ 2.5 wt% in the range up to 120 °C is due to the evaporation of moisture and low molecular weight impurities. In the range from 130 to 350 °C, a more visible weight loss of ~ 5.5 wt% is observed, which is associated with the release of the residual solvent NMP that forms donor–acceptor bonds with polymers of heteroaromatic structure, which complicates its total removal from the membranes^54^*.* In the range of 370–400 °C, the weight loss associated with the thermal degradation of the polymer chains begins.

**Figure 6:**
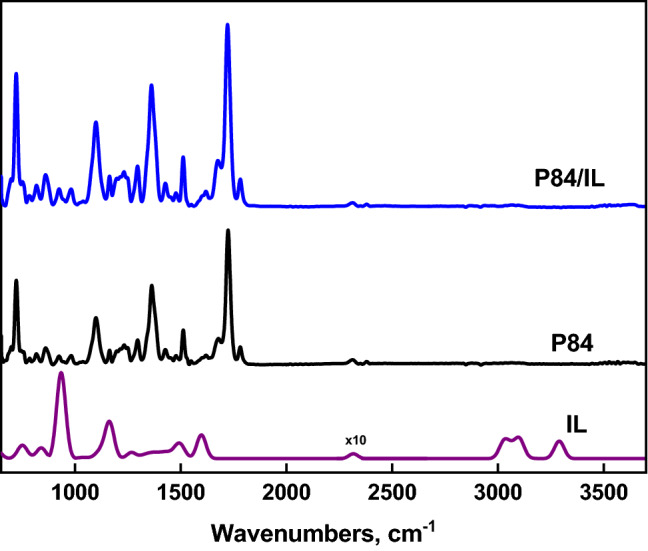
ATR-FTIR spectra of IL, P84 and P84/IL membranes.

To study the chemical composition of the membranes, ATR-FTIR spectroscopy was used. Figure 6 shows the IR spectra of the ionic liquid (IL), P84 and P84/IL membranes. Typical imide bands for P84 are located at 1782 cm^−1^ for symmetric CO stretching, at 1716 cm^−1^ for asymmetric CO stretching, and at 1359 cm^−1^ for CN stretching. The IL characteristic bands at 3158 cm^−1^ correspond to the CH stretching vibration within the imidazole ring. For the IL spectrum, it is worth noting the peak at 2224 cm^−1^ assigned to the interaction of ions within the molecule, as well as the bands in the range 1000–750 cm^–1^ corresponding to the anion, and 936 cm^–1^ assigned to B-C stretching^55^. Thus, the IL characteristic bands are not present in the spectrum of P84/IL membrane. Therefore, it can be concluded that the P84/IL membrane does not contain an ionic liquid.

### Membrane structure

**Figure 7:**
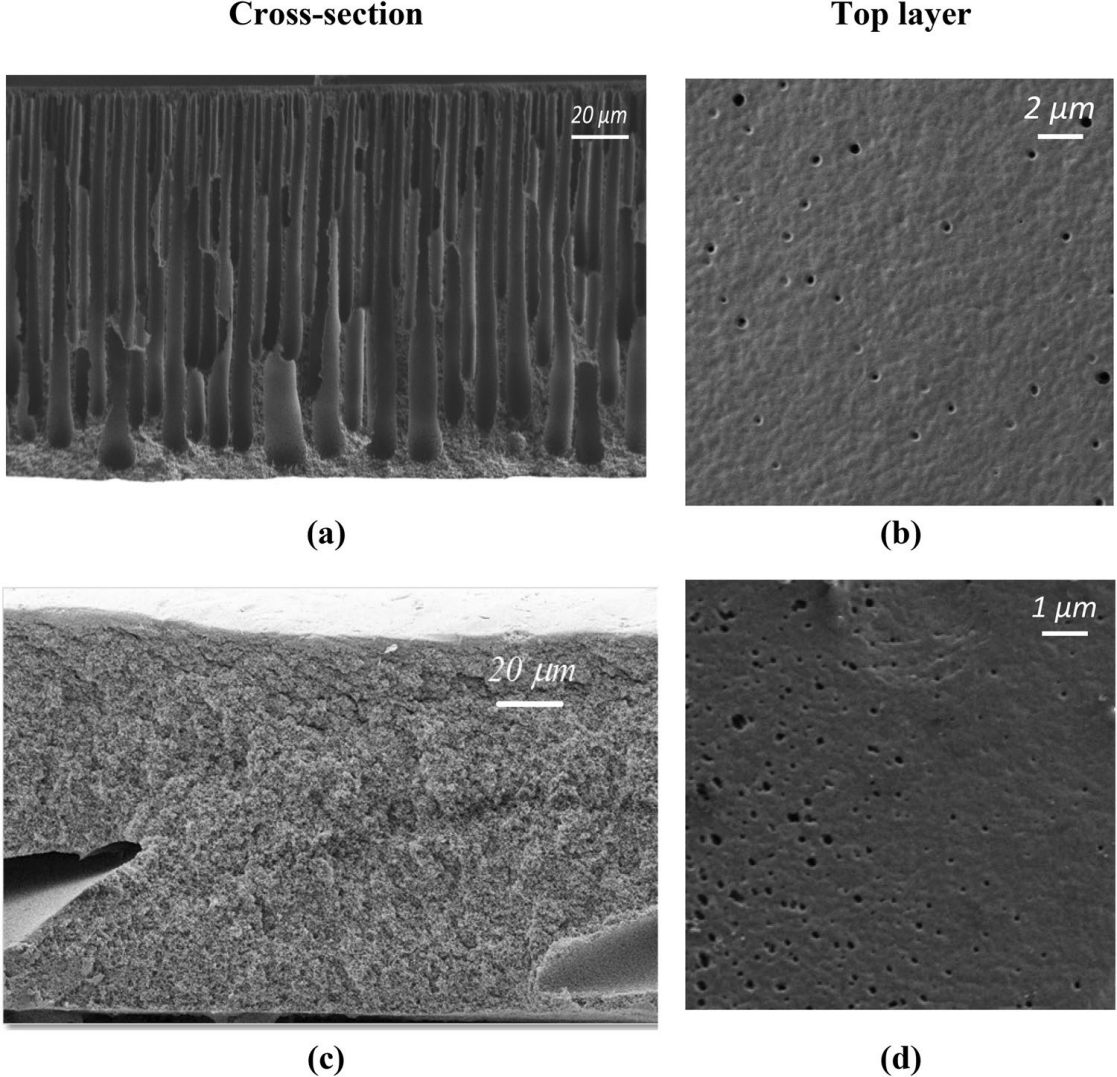
SEM micrographs of the membrane cross-section and top surface for (**a**,**b**) the P84 and (**c**,**d**) P84/IL membranes.

Scanning electron microscopy (SEM) was used to study the membrane morphology. Figure 7 shows micrographs of cross-section and top surface for the P84 and P84/IL membranes. The cross-sections of both membranes have an anisotropic structure consisting of a thin top layer and a porous substrate that is typical for UF membranes. Figure 7a shows cross-section of the P84 membrane, where the substrate exhibits finger-like porous structure. For P84/IL membrane, the structure of the cross-section changes significantly. Figure 7c demonstrates a spongy structure of the porous substrate for the P84/IL membrane. Such structure is formed during phase inversion process which occurs more slowly after IL addition in view of a significant increase in the dynamic viscosity of the polymer casting solution. Figure 7d shows that the IL addition as a co-solvent leads to an increase in the number of pores in the top layer, as well as to the formation of cavities in the P84/IL membrane structure.

### Transport properties

**Figure 8:**
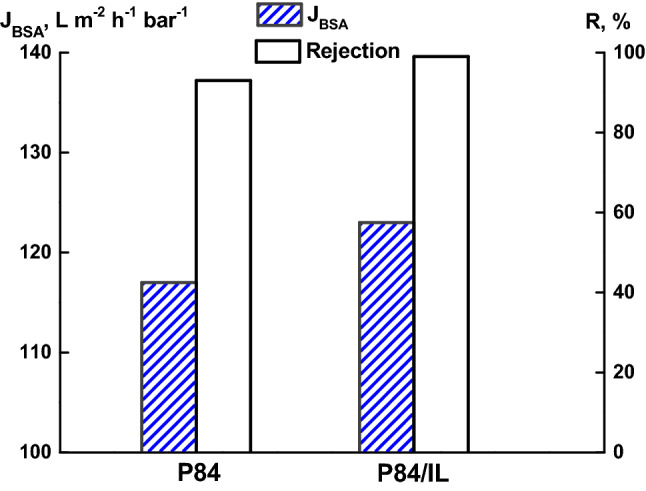
The flux of aqueous solution of BSA (*J*_*BS*A_) and the BSA rejection (*R*) for the P84 and P84/IL membranes.

UF tests with pure water and an aqueous solution of bovine serum albumin (BSA) were performed to evaluate the transport properties of P84 and P84/IL membranes prepared from 15 wt% polymer solutions. Figure 8 shows the data on the flux and the rejection that were determined by filtrating an aqueous solution of BSA through the P84 and P84/IL membranes. The use of IL as a co-solvent leads to an increase in membrane performance; the BSA rejection for the studied membranes is very high (93–99%).

**Figure 9:**
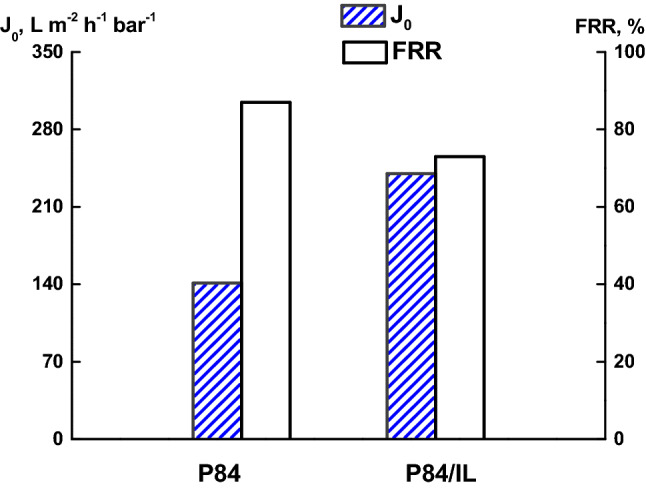
The pure water flux (J_0_) and the flux recovery ratio (FRR) for the P84 and P84/IL membranes.

Figure 9 shows the data on the pure water flux and the flux recovery ratio *(FRR*) for the P84 and P84/IL membranes. The IL addition to the casting solution increases the hydraulic performance of the membranes. The *FRR* for the membranes based on P84 is high enough; however, in the case of P84/IL membrane, its value slightly decreases.

**Figure 10:**
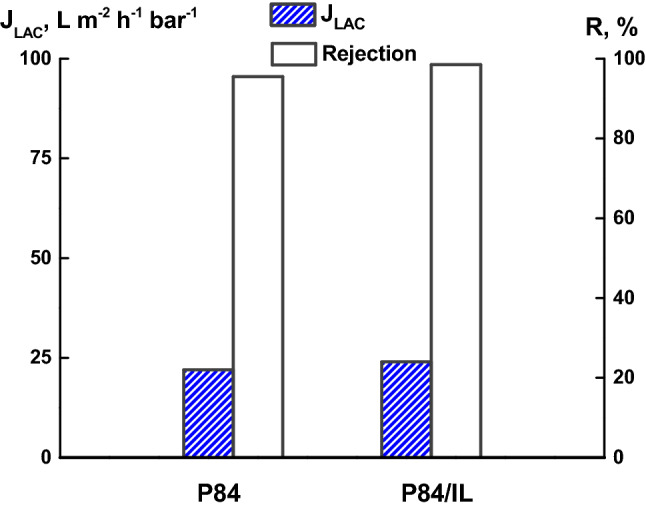
The flux of LAC in water–acetone solution (J_LAC_) and the LAC rejection (R) for the P84 and P84/IL membranes.

The rejection capacity of P84 and P84/IL membranes prepared from 15 wt% polymer solutions was evaluated with respect to La^3+^ in the form of a lanthanum-alizarin complex (LAC). Figure 10 shows the results on filtration of LAC in aqueous acetone solution in the form of the LAC flux (*J*_*LAC*_) and LAC rejection (*R*) for the P84 and P84/IL membranes. Both membranes are characterized by a high rejection of 96–98%, as well as high flux. At the same time, the P84/IL membrane exhibits a slightly higher rejection compared to the P84 membrane. The success of this experiment results from the stable performance (operability) of the P84-based UF membranes, which are stable for filtering not only aqueous solutions, but also aqueous acetone media.

Thus, the obtained results demonstrate the possibility of efficient extraction of the rare-earth metal from dilute solutions using the UF membranes based on P84 copolyimide, including those with IL additives.

## Conclusions

The effect of 1-hexyl-3-methylimidazolium tetracyanoborate ([hmim][TCB]) additives on the structure and transport properties was studied for the P84/IL asymmetric membrane prepared from a P84/IL/NMP casting solution in the ratio of 15/15/70 (wt%). The P84/IL asymmetric membrane was compared to the P84 membrane prepared from a P84/NMP casting solution in the ratio of 15/85 (wt%). A significant difference in the structures of the porous substrates was revealed by SEM on the membrane cross-sections. The P84 membrane has finger-like structure of the porous substrate, in contrast to spongy structure of the substrate for P84/IL membrane. Such significant change in morphology is due to an increase in the dynamic viscosity of the casting solution containing IL, which decreases the rate of the phase inversion process; in addition, the IL leaks when it is contained in the coagulation bath. According to FTIR, the P84/IL membrane does not contain an ionic liquid.

The transport parameters of the membranes were determined in the UF tests with pure water and an aqueous solution of bovine serum albumin (BSA). It was shown that the IL addition to the P84 casting solution increases the performance of the membranes. The FRR for the membranes based on P84 is quite high; however, its value decreases slightly for the P84/IL membrane.

The rejection capacity of the P84 and P84/IL membranes was evaluated with respect to La^3+^ in the form of a lanthanum-alizarin complex (LAC) in aqueous acetone solution. The P84/IL membrane showed a high rejection (98.5%) with respect to LAC, as well as a high flux. Thus, the obtained results allow us to conclude that it is possible to extract efficiently the rare earth metal from dilute solutions using the ultrafiltration membranes based on P84 copolyimide, including those with IL additives.

## Supplementary Information

Supplementary Table S1.

## Data Availability

All data generated and analysed during this study are included in this published article and its supplementary information files.
