# Supplementary material for: Effect of ionic liquid on formation of copolyimide ultrafiltration membranes with improved rejection of La3+
Source: Sci Rep. 2022 May 17;12:8200. doi: 10.1038/s41598-022-12377-0 (PMC9114424; doi:10.1038/s41598-022-12377-0)
Supplement: Supplementary file 1 — Supplementary Table S1. [file 41598_2022_12377_MOESM1_ESM.docx]

**Effect of Ionic Liquid on formation of copolyimide ultrafiltration membranes with improved rejection of lanthanum (3+)**

**Supplementary information**

Alexandra Pulyalina ^1,*^, Konstantin Grekov ^2^, Vera Tataurova ^1^, Anna Senchukova ^1^, Alexander Novikov ^1^, Ilya Faykov ^1^, and Galina Polotskaya ^1,3^

^1^ Institute of Chemistry, Saint Petersburg State University, Saint Petersburg, 198504, Russian Federation
^2^ Тhe Bonch-Bruevich St. Petersburg State University of Telecommunications, Saint Petersburg, 193232, Russian Federation
^3^ Institute of Macromolecular Compounds, Russian Academy of Sciences, Saint Petersburg, 199004, Russian Federation
^*^ a.pulyalina@spbu.ru

**Table S1**. Calculated enthalpies, entropies, and Gibbs free energies (in Hartree) for optimized equilibrium model structures (H, S, and G, respectively).

| **Optimized equilibrium model structure** | **H, kcal/mol** | **S, kcal/mol** | **G, kcal/mol** |
| --- | --- | --- | --- |
| N-methylpyrrolidone | -325.696987 | 81.446 | -325.735684 |
| ionic_liquid_cation---N-methylpyrrolidone | -748.538125 | 147.994 | -748.608441 |
| ionic_liquid_cation | -422.807624 | 103.645 | -422.856869 |
| ionic_liquid_anion---N-methylpyrrolidone | -721.898103 | 142.105 | -721.965621 |
| ionic_liquid_anion | -396.189386 | 92.344 | -396.233261 |
| P84---N-methylpyrrolidone | -2273.910977 | 269.458 | -2274.039005 |
| P84---ionic_liquid_cation | -2371.040065 | 284.405 | -2371.175195 |
| P84---ionic_liquid_anion | -2344.409830 | 286.031 | -2344.545733 |
| P84 | -1948.193366 | 233.839 | -1948.304471 |
